# Association between infection and nutritional status among infants in a cohort study of vitamin A in western Kenya

**DOI:** 10.3389/fnut.2022.921213

**Published:** 2022-09-23

**Authors:** Frederick K. Grant, Rose Wanjala, Jan Low, Carol Levin, Donald C. Cole, Haile S. Okuku, Robert Ackatia-Armah, Amy W. Girard

**Affiliations:** ^1^International Potato Center, Dar es Salaam, Tanzania; ^2^International Potato Center, Nairobi, Kenya; ^3^Department of Global Health, University of Washington, Seattle, WA, United States; ^4^Dalla Lana School of Public Health, University of Toronto, Toronto, ON, Canada; ^5^International Potato Center, Kigali, Rwanda; ^6^Rollins School of Public Health, Emory University, Atlanta, GA, United States

**Keywords:** inflammation, infection, vitamin A deficiency, iron deficiency, infants

## Abstract

**Background:**

Infection is associated with impaired nutritional status, especially for infants younger than 5 years.

**Objectives:**

We assessed the impact of infection indicated by both acute phase proteins (APP), C-reactive protein (CRP), and α-1-acid-glycoprotein (AGP), and as reported by maternal recall on the nutritional status of infants.

**Materials and methods:**

A total of 505 pregnant women were enrolled in a nested longitudinal cohort study of vitamin A (VA). Data from 385 children are reported here. The incidence and severity of respiratory infection and diarrhea (previous 14 days) were assessed by maternal recall; infant/child feeding practices were collected. Infant weight, recumbent length, and heel-prick capillary blood were taken at 9 months postpartum. Indicators of the VA status [retinol binding protein (RBP)], iron status (Hb, ferritin), and subclinical inflammation APP, CRP (>5 mg/L), and AGP (>1 g/L) were determined. Impacts of infection on the infant nutritional status were estimated using logistic regression models.

**Results:**

Infection prevalence, based on elevated CRP and AGP levels, was 36.7%. For diarrhea reported symptoms, 42.4% of infants at 9 months had no indication of infection as indicated by CRP and AGP; for acute respiratory reported symptoms, 42.6% had no indication of infection. There was a significant positive association with infection among VA-deficient (RBP < 0.83 μmol/L) infants based on maternal reported symptoms but not with iron deficiency (ferritin < 12 μg/L). The odds of having infection, based on increased CRP and AGP, in underweight infants was 3.7 times higher (OR: 3.7; 95% CI: 2.3, 4.5; *P* = 0.019). Infants with iron deficiency were less likely (OR: 0.40; 95% CI: 0.1, 0.7; *P* = 0.001) to have infection based on CRP and AGP, while infants with VA deficiency were five times more likely (OR: 5.06; 95% CI: 3.2, 7.1; *P* = 0.0001) to have infection.

**Conclusion:**

Acute phase proteins are more useful in defining infection in a population than reported symptoms of illness. Not controlling for inflammation in a population while assessing the nutritional status might result in inaccurate prevalence estimation.

## Introduction

An inflammatory response is biochemical and physiological changes that occur in the body as a result of an infection (1). An infection may, however, occur without inflammation, and inflammation may also develop due to non-infectious causes (2). Infection or the inflammation process is accompanied by an acute phase response, a non-specific process that includes the production of acute phase proteins such as C-reactive protein (CRP), and α-1-acid glycoprotein (AGP) (3).

Production of CRP and AGP is useful in the screening of diseases, monitoring the response of treatment, and detection of incurrent infections (1, 4–6). During an acute phase response, the level of CRP increases drastically within 6–8 h following the onset of infection, peaking as much as 1,000 times more than the baseline values within 48 h and then declining afterward (7–10). Whereas, CRP concentration levels rise to a maximum within 24–48 h following infection, AGP responds more slowly and can take up to 3–5 days to reach plateau and then remains elevated for a longer period as the CRP level declines (8, 11, 12). In a population, a combination of AGP and CRP is useful in monitoring infection not only in symptomatic individuals but also in monitoring infection in asymptomatic or healthy individuals. It can detect those who have been recently infected with no clinical symptoms (elevated CRP) and those who are recovering and convalescing (high AGP and low CRP) (1, 13).

Plasma concentrations of nutritional markers such as retinol, iron, ferritin, and transferrin receptor are influenced by inflammation and infection (12–18). The relationship between vitamin A deficiency and infection in children are bidirectional, with each increasing the risk of the other (7, 19). Infection causes endogenous consumption of macronutrients and alterations of many micronutrients and nutrition markers (1). It decreases intestinal absorption of nutrients, thus resulting in loss of nutrients. During the acute phase response, indicators of iron, zinc, and vitamin A statuses can be altered, leading to inaccurate estimations of deficiency prevalence in populations with high or unknown prevalence of infections (20). Vitamin A deficiency is commonly estimated using serum or plasma retinol concentrations, but retinol is reduced by clinical and subclinical infections; thus, the proxy measure can result in overestimation. Thurnham et al. (13) showed that after adjusting for inflammation, the estimates of vitamin A deficiency in healthy individuals with no raise in acute phase proteins were the same as those obtained by adjustment of plasma retinol concentrations in the whole group using acute phase proteins. In Ghana, a study found that serum retinol was not associated with symptoms of illness but showed correlation with acute phase proteins AGP and serum amyloid A (20).

We previously showed a high prevalence of subclinical inflammation (33%), and iron (27–83%) and vitamin A (20%) deficiencies among children in this population of western Kenya (18, 21–23). Children with a poor vitamin A status tend to suffer from more severe and prolonged episodes of diarrhea (7). The association of diarrhea and low serum retinol levels was reported in Peru, where findings suggested that diarrhea might lead to lower circulating retinol concentration and perhaps to its depletion (24). In Nepal, children with a history of diarrhea or respiratory diseases were found to have low levels of serum retinol and retinol-binding protein, and children with mild vitamin A deficiency are at a greater risk of respiratory diseases or diarrhea (25). Morbidity by reported illness or the presence of infection as indicated by acute phase protein concentrations was associated with low serum retinol (25).

The Sweetpotato Action for Security and Health in Africa (Mama SASHA) project was a 5-year cluster-allocated community-based proof-of-concept study that integrated agriculture, nutrition, and health to improve the nutritional status of pregnant women and their children in western Kenya (26). The cohort study was a nested prospective longitudinal study within the Mama SASHA community-based study with the objective of assessing how the uptake of the orange-fleshed sweet potato (OFSP) interventions including knowledge, farming, and consumption of OFSP impacted the vitamin A, nutrition, and health statuses of mothers and their infants from mid-pregnancy to 9 months postpartum (27). The goal of the analysis reported here was to assess the association between infection as indicated by the acute phase proteins, CRP and AGP, and as reported by maternal recall and the nutritional status of infants.

## Materials and methods

### Study design and population

A full description of the Mama SASHA program including its development and design, activities, and monitoring evaluation strategy, is presented elsewhere (26). The cohort study of vitamin A (COVA) was one of the evaluation strategies of the Mama SASHA program (26). This was a nested community-based longitudinal cohort study that enrolled pregnant women in their early pregnancy (10–24 weeks) and followed them up to 9 months postpartum. The study was implemented across eight health facilities from October 2012 to June 2014 in Bungoma and Busia counties, western Kenya; four of the health facilities were based in the intervention areas where OFSP activities were carried out, while the other four facilities were located in the control areas, with no OFSP activities (27).

### Inclusion and exclusion criteria

Pregnant women were included in the study if they were between the age of 17 and 40 years, attending their first ANC visit, and between gestational age of 10 and 24 weeks as determined either by maternal report of the last menstrual period or by palpations performed by the nurse at the ANC. The mother should have intentions to breastfeed and to reside in the catchment area until the child was 10 months old. Exclusion criteria included previous involvement in the Mama SASHA intervention during earlier pregnancy. In the intervention facilities, women were excluded if they did not live in a Mama SASHA intervention village. While in the control facilities, women were excluded if they resided in a Mama SASHA intervention village.

### Sample size estimation

From the 2010 baseline household survey data on predictors of child VA status conducted in Mama SASHA implementation communities, we estimated the intraclass correlation coefficient for the infant VA status to be 0.02 (26). Data on RBP in women were not available for this population. However, assuming a population SD in breast milk retinol of 0.7 mmol/L (27), we estimated that 400 women would be required to detect a significant double difference in breast milk retinol of 0.1 mmol/L, with 95% confidence and 80% power. Recruiting an additional 25% for anticipated losses to follow-up yielded an estimated required sample size of 500 women.

### Data collection

Data collection occurred at four time points over the course of the study: at enrollment (10–24 weeks gestation); in the last trimester, approximately 4–6 weeks prior to delivery; at 4 months; and at 9 months postpartum. Prior to the implementation of the study, community sensitizations and training of all stakeholders were carried out. The community health workers (CHWs) played a crucial role in the recruitment and follow-up of the participants throughout the study period. The nurses carried out recruitment at the antenatal clinics in the participating facilities assisted by a trained CHW. They administered a standardized pre-screening tool, and if eligibility criteria were met, the nurses would introduce the study and refer the pregnant woman to the COVA study offices. Following further screening by research assistants, a written informed consent was obtained. All research protocols were approved by the institutional review boards at Emory University and the Kenya Medical Research Institute. Standardized questionnaires were then administered by trained research assistants to collect detailed information on sociodemographic characteristics, household food security and diet diversity, maternal and infant diet and consumption of VA-rich foods, maternal health and nutrition knowledge, sweetpotato production, and participation in program activities. Data verification was carried out in Census and Survey Processing System Software (CSPro), version 6.0 (US Census Bureau and Inner-City Fund International).

**FIGURE 1:**
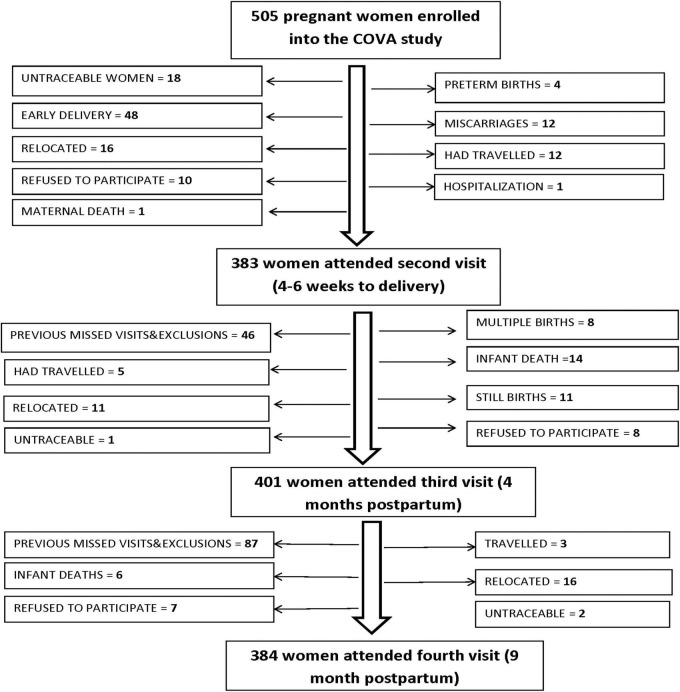
Numbers and reasons for loss to follow up for the 505 women enrolled in the cohort study of vitamin A (COVA) in western Kenya.

Of the 505 women enrolled in the study, 383 women returned for their second visit at late pregnancy, with 401 and 385 women attending the third visit at 4 months postpartum and the fourth visit at 9 months postpartum, respectively (Figure 1). Overall, a 26.4% loss to follow-up was experienced in the course of the study. We assessed bias due to loss to follow-up by comparing mothers completing the fourth visit and those lost to follow-up.

Data analysis in this study was limited to infant data at 9 months postpartum. During this visit, the incidence and severity of respiratory infection and diarrhea over the previous 14 days were assessed by maternal recall. Information on infant feeding practices was also collected, and child feeding practices were assessed using WHO-recommended infant and young child feeding (IYCF) indicators. The infant birth weight was collected as soon as possible after birth and at most within 1 week of birth. Research assistants collected the birth weight if the mother delivered at the facility or by trained community health workers in cases of delivery at home. Birth weight was measured with the mother holding the infant and using a tarred Seca 874 mother baby scale accurate to 10 g. Infant weight, recumbent length—measured to the nearest millimeter using a Schorr measuring board (weigh and measure, MD), and blood collection by heel prick were measured at 9 months postpartum. At 9 months, infant weight was taken by tarring the scale to zero with the mother still on the scale, and her child was handed to her to hold. The digital readout would indicate the child’s weight only. The length of the child was obtained using a Schorr recumbent length board (Weigh and Measure, MD). An enumerator would place the child lying recumbent on the board and assure proper placement; the mother of the child would assist in assuring the child remains still, while the second enumerator recorded the length reading read out by the first enumerator. Each enumerator took two measurements from the same child; if the two measurements differed by more than 0.1 cm, a third measurement was taken. Data were entered immediately using Emergency Nutrition Assessment software, and errors detected and corrected.

Research assistants obtained heel-prick capillary blood samples between 0800 and 1600 h by using single-use sterile Contact-Activated BD Microtainer Lancets (Becton, Dickinson and Company, Franklin Lakes, NJ). Blood was collected in BD Microtainer tubes with EDTA (Becton, Dickinson and Company, Franklin Lakes, NJ). Hemoglobin was determined within 3 min of blood collection with the use of a HemoCue 201+ system (Radiometer Medical Aps, Copenhagen, Denmark) machine and adjusted for altitude (28). An additional 400–500 ml capillary blood was collected into BD Microtainer tubes with EDTA and, within 12 h of blood collection, centrifuged at 1,500 × g for 5 min at 27^°^C. Plasma was then removed and stored in Nalgene cryogenic tubes (Thermo Fisher Scientific, Waltham, Massachusetts) at −20^°^C in solar-powered freezers at the health facility until collected and transported on ice for longer term storage at the KEMRI/CDC Malaria Laboratory in Kisumu. The samples were transported on dry ice to Germany for subsequent laboratory analysis of retinol-binding protein (RBP), C-reactive protein (CRP), and α-1-acid glycoprotein (AGP) by using a sandwich ELISA technique (29, 30). All of the samples were measured in duplicate, and the intra- and inter-assay CVs were <10%. A low infant RBP status, as a proxy for vitamin A deficiency, was defined as an RBP <0.83 μmol/L on the basis of cutoffs derived from a nationally representative survey in infants in Cameroon with similar a background prevalence of inflammation (31, 32). Anemia was defined as an altitude-adjusted hemoglobin of <110 g/L in infancy (28). The threshold for iron deficiency in infants using a low ferritin level was ferritin <12 μg/L (33).

### Statistical analysis

The distributions of the various biomarker concentrations were assessed for normality by the use of normality plots and Kolmogorov–Smirnov tests. The distributions of ferritin, RBP, CRP, and AGP were non-Gaussian and thus were log-transformed and reported as geometric means and SDs. Vitamin A deficiency and iron deficiency were adjusted for the effect of inflammation with the use of a correction factor approach to adjust values of RBP and ferritin on the basis of the elevated acute phase proteins CRP and AGP, as described elsewhere (18, 33). Correction factors for RBP and ferritin were as follows: 1.08 and 0.77, respectively, for the incubation stage (elevated CRP and normal AGP), 1.46 and 0.35, respectively, for early convalescent inflammation (elevated CRP and AGP); and 0.70 and 0.75, respectively, for late convalescent inflammation (elevated AGP and normal CRP). Anthropometric measures were calculated using the WHO child growth standards for underweight (weight-for-age *Z*-score < −2 SD), stunting (height- and length-for-age *Z-*score < −2 SD), and wasting (weight-for-height and -length *Z-*scores < −2 SD) (34). The primary outcome, infection, was assessed using both the acute phase proteins, CRP and AGP, and maternal reported morbidity symptoms from recall. In total, four categories were created: no infection (CRP ≤ 5 mg/L and AGP ≤ 1 g/L), incubation (CRP > 5 mg/L and AGP ≤ 1 g/L), early convalescence (CRP > 5 mg/L and AGP > 1 g/L), and late convalescence (CRP ≤ 5 mg/L and AGP > 1 g/L). Having an infection meant being in incubation or early convalescence or late convalescence. Infection based on maternal recalls was defined as having reported symptoms of diarrhea, acute respiratory infection (ARI), fever, and vomiting. ARI was defined as a child having cough or cough plus runny nose, coughing, wheezing, or experiencing rapid breathing in the past 14 days. Diarrhea, fever, and vomiting were measured as having any of the respective symptom in the past 14 days.

For all categorical variables, a chi-square test was used to determine the differences. Student’s *t*-test was used for all continuous variables to compare the means. The cluster randomized longitudinal approach employed in the COVA study, while robust and efficient for assessing differences in change over time due to the Mama SASHA program (i.e., time–intervention interaction), presents analytic challenges, such as clustering and potentially unmeasured or random effects at the cluster level—in this case, the village and facility (26, 27). In the case of the COVA study, we have at least four levels of clustering, the first being repeated measurement occasions (level 1 units) (this level of clustering, i.e., repeated measurements were not considered in the analysis of this current study). The other levels of clustering were the individual participants (*n* = 385 at 9 months postpartum, level 2) which are nested within facilities (level 3 units) and villages (level 4). Ignoring clustering at any of these two levels and potential random effects introduced at the *facility* or *village* level can result in the incorrectly specified estimates effect (34, 35). Therefore, a random effect multivariate logistic regression model was performed to assess the association between the nutritional status and infection based on both maternal reported symptoms and elevated levels of AGP and CRP controlling for birth weight, age and gender of infant, food insecurity, and wealth. All statistical analyses were performed using STATA 13.1 (StataCorp, College Station, TX) software and SAS 9.4 (SAS Institute Inc., Cary, North Carolina) software. Significance was defined as *P* < 0.05.

## Results

The overall mean age of the women enrolled was 24.3 years. About half (48%) of the women had attained primary education, and 31% had no education. Maternal nutritional and health knowledge was low in this population. One-third (34%) responded to having heard of vitamin A, 27% had knowledge that vitamin A prevents disease, and 19% were aware that vitamin A protects eyesight (Table 1).

**TABLE 1:** Sociodemographic and birth characteristics of 385 women participating in the Mama SASHA cohort study in western Kenya.^a^

Characteristic	
Maternal age, years	24.3 ± 5.5
Gestational age in weeks	20.4 ± 5.1
Maternal Education, completed category	
<Primary	118 (30.7)
Primary	186 (48.3)
Above primary	81 (21.0)
Maternal marital status^b^	
Single	53 (13.7)
Married/partnered monogamous	304 (79.0)
Polygamous	27 (6.9)
Maternal occupation	
Does not work	153 (39.8)
Agriculture	129 (33.4)
Other (petty trading, government work, etc.)	103 (26.8)
Household food insecurity category	
Mild/secure	215 (55.8)
Moderate	80 (20.8)
Severe	90 (23.4)
Household dietary diversity score	5.45 ± 1.42
Mother has heard of vitamin A	131 (33.9)
Mother knows VA prevents disease	106 (26.9)
Mother knows that VA protects the eyes	72 (18.7)
Household asset index score	8.55 ± 1.77
HH harvested SP in past 12 months	141 (36.6)

^a^Values are means ± SD, n = 385, or percent values within parentheses.

^b^Missing value for marital status, n = 1.

There was low prevalence of stunting, wasting, and underweight among this sample of children at 9 months of age. The prevalence of iron deficiency, as indicated by ferritin levels, was 41.6%; vitamin A deficiency, as indicated by RBP levels, was also high at 25.6%. Elevated CRP and AGP were present in 18 and 38% of infants at 9 months, respectively. The prevalence of reported acute respiratory infections was high at 9-month visits (Table 2).

**TABLE 2:** Demographic, infection, and biochemical characteristics in a sample of 9-month-old Kenyan children.^a^

Characteristics	
Birth weight, *kg* (*n* = 385)	3.27 ± 0.05
Infant WAZ score (*n* = 372)	−0.24 ± 0.99
Infant LAZ score (*n* = 372)	−0.70 ± 1.06
Infant WLZ score (*n* = 372)	0.26 ± 1.17
% stunted (<-2 SD)	39 (10.5)
% wasted (<-2 SD)	8 (2.2)
% underweight (<-2 SD)	14 (3.8)
RBP, μmol/L (*n* = 375) ^b^	1.74 ± 1.5
Low RBP (< 1.17 μmol/L) *n* = 375) ^b^	96 (25.6)
Plasma ferritin (*n* = 375) ^b^	19.56 ± 16.3
Iron deficiency (ferritin < 12 μg/L) (*n* = 375) ^b^	156 (41.6)
CRP (>5 mg/L, *n* = 375),%	68 (18.1)
*CRP, mg/L* (= 375)	4.06 ± 8.4
AGP (> 1.0 g/L, *n* = 375),%	143 (38.1)
AGP, g/L (*n* = 375)	0.99 ± 0.57
Had diarrhea in past 14 days	29.5 (15.3)
Mean number of days had diarrhea	3.74 ± 3.0
Had blood or mucus in stool	13 (44.6)
Had Prolonged diarrhea (>10 days)	2 (0.8)
Had acute respiration infection	58 (29.9)
Had fever	40 (20.8)
Had vomiting	14 (7.0)

^a^Values are means ± SD, n = 385, or percent values within parentheses. LAZ, length-for-age Z-score; RBP, retinol-binding protein; WAZ, weight-for-age Z-score; WLZ, weight-for-length Z-score.

^b^RBP and ferritin adjusted for inflammation using the Thurnham correction factor method (32).

Overall, the prevalence of infection in the infants based on elevated CRP and AGP levels was 36.7%, with 3.32% at the incubation stage, 14.3% at early convalescence, and 18.9% at late convalescence.

Table 3 compares infection based on CRP and AGP levels to maternal recalls of symptoms in the past 14 days. For diarrhea reported symptoms, 42.4% of infants had no indication of infection, while for acute respiratory reported symptoms, 42.6% had no indication of infection as measured by CRP and AGP levels. Among those with reported fever, 60% had infection, while among those with vomiting, 63% had infection as measured by CRP and AGP levels. The results of a multivariate logistic regression model showed that the odds of infection with the nutritional status did not differ when inflammation was adjusted for or not, using maternal recalls as an indication of the infection status. However, there was a significant positive association with infection among vitamin A-deficient infants based on maternal reported symptoms (Table 4). A non-significant (*p* = 0.304) inverse association was found among underweight infants with infection, with those being underweight not likely to have infection when using reported illness to define the infection status.

**TABLE 3:** Differences in infection as indicated by acute phase proteins (CRP and AGP) vs. maternal recalls of symptoms in the past 14 days at 9 months postpartum.^a^

	Elevated CRP and AGP		*P* ^b^
Symptom reported	Infection	No infection	
**Diarrhea**			
Yes	34 (57.6)	25 (42.4)	**0.004**
No	123 (37.7)	203 (62.3)	
**Acute respiratory infections**			
Yes	66 (57.4)	49 (42.6)	**0.0001**
No	91 (33.7)	179 (63.3)	
**Fever**			
Yes	48 (60.0)	32 (40.0)	**0.0001**
No	109 (37.7)	196 (64.3)	
**Vomiting**			
Yes	17 (63.0)	10 (37.0)	0.150
No	140 (39.1)	218 (60.9)	

^a^Values are n = 385 or percent values within parentheses.

^b^P-values for differences in the infection status as indicated by acute phase proteins or maternal recall were calculated using a complex survey chi-square test.

The bold values are significant at p < 0.05.

**TABLE 4:** Multivariate analysis^a^ of odds for maternal symptom recall association with infant biological nutritional status in the COVA study.^b^

Nutritional status variables	Adjusted odds ratio^c^	*P* (Wald test)
Stunting (LAZ < −2 SD)	1.02 (0.4, 2.3)	0.932
Wasting (WLZ < −2 SD)	1.66 (0.6, 2.8)	0.463
Underweight (WAZ < −2 SD)	0.56 (0.2, 1.2)	0.304
Iron deficiency (ferritin < 12 μg/L)^d^	1.25 (0.3, 2.4)	0.344
Vitamin A deficiency (RBP < 0.83 μmol/L)^d^	1.67 (1.3, 2.1)	**0.006**

^a^Controlling for birth weight, age, and gender of infant; food insecurity; and wealth.

^b^Infection based on maternal recall is defined as maternal reported incidence of diarrhea and/or respiratory illness of infant in the past 14 days.

^c^All values within parentheses are 95% CIs.

^d^RBP and ferritin adjusted for inflammation using the Thurnham correction factor method (32).

The bold values are significant at p < 0.05.

When employing elevated levels of CRP and AGP to define the infection status, the odds of having infection in the underweight infants was 3.7 times higher (Table 5). Without adjusting for inflammation (in assessing ferritin levels as an indicator of the iron status), infants with iron deficiency were less likely to have infection (*OR* = 0.40; 95% CI: 0.1, 0.7; *P*-value = 0.001), but after adjusting for inflammation (in assessing ferritin levels), the association was null (*OR* = 1.11; 95% CI: 0.7, 1.9; *P*-value = 0.607). Vitamin A deficiency was found to be significantly associated with infection as indicated by CRP and AGP levels. Without adjusting for inflammation, we found that infants with vitamin A deficiency were five times more likely to have infection (*OR* = 5.06; 95% CI: 3.2, 7.1; *P*-value = 0.0001), but after adjusting for inflammation, they were significantly less likely to have infection (*OR* = 0.55; 95% CI: 0.3, 0.8; *P*-value = 0.002).

**TABLE 5:** Multivariate analysis^a^ of odds for inflammation marker (elevated CRP and AGP) association with infant nutritional status in the COVA study.

Nutritional status variables	Adjusted odds ratio^b^	*P* (Wald test)
Stunting (LAZ < −2 SD)	1.59 (1.1, 2.6)	0.057
Wasting (WLZ < −2 SD)	1.51 (0.7, 2.4)	0.535
Underweight (WAZ < −2 SD)	3.71 (2.3, 4.5)	**0.019**
Iron deficiency (ferritin < 12 μg/L)^c^	0.40 (0.1, 0.7)	**0.001**
Vitamin A deficiency (RBP < 0.83 μmol/L)^c^	5.06 (3.2, 7.1)	**0.0001**

^a^Controlling for birth weight, age, and gender of infant; food insecurity; and wealth.

^b^All values within parentheses are 95% CIs.

^c^RBP and ferritin adjusted for inflammation using the Thurnham correction factor method (32).

The bold values are significant at p < 0.05.

## Discussion

The prevalence of subclinical inflammation as indicated by acute phase proteins, CRP or AGP, was high in this sample of 9-month-old infants in western Kenya, similar to prior reports in this setting (18). Our findings support the importance of using acute phase responses such as CRP and AGP as objective indicators of acute infection status in capturing episodes of infection in children, rather than caregiver morbidity recalls in previous days, which can be subjective and thus unreliable due to potential recall bias (10). We found that approximately 50% of the infants reported to have symptoms of diarrhea or ARI or fever based on maternal recall in the past 14 days had no infection as indicated by elevated CRP and AGP levels (Table 3). While assessing if there was any association between reported infection (defined as having diarrhea, ARI, fever, or vomiting) and nutritional status of the infants, we found no significant differences, except for vitamin A deficiency. Based on our findings, there was no association between reported infection and nutritional status with or without inflammation adjustment when using reported symptoms to define inflammation. However, when using the acute phase proteins as indicators for acute infection, there was a significant association between infant nutritional status and infection. Thus, the use of reported symptoms to assess the nutritional status might lead to either overestimation or underestimation of the nutritional status in a population. These findings support results from a complex study in Nepal assessing the relation between infection, and acute phase proteins and serum retinol protein in respect to night blindness (7). Using the elevated levels of CRP and AGP helps in capturing infection missed out by the maternal reported symptoms as well as asymptomatic infants, who show no symptoms but have subclinical infection.

Our findings also highlight the importance of adjusting for infection and inflammation in a nutritional intervention. We previously reported that correcting for inflammation, based on CRP and AGP levels, modified the estimated levels of the iron status and prevalence of iron deficiency among pre-schoolers in a similar setting of western Kenya (18). Similar studies indicated similar changes in the vitamin A status and deficiency in resource-poor settings (13, 15). This is because many iron and vitamin A status indicators are influenced by inflammatory processes, and not controlling for such influence will result in inaccurate estimation of true micronutrient (such as iron and vitamin A) statuses at the population level. Without controlling for inflammation, infants with vitamin A deficiency were five times more likely to have infection, but when corrected for inflammation, the odds of having infection was less by 45% than the odds of those with no vitamin A deficiency. Adequate vitamin A stores have been associated with the maintenance of the integrity of the epithelial barrier and also positively associated with measures of innate immune activity, suggesting a protective effective against different pathogens (36, 37). Without correcting for inflammation, a negative association is observed with iron deficiency, but when we corrected for inflammation, there was a positive association with infection among infants with iron deficiency as compared to those with no iron deficiency (Table 5). During an acute phase inflammation, iron is redistributed into the liver and mononuclear phagocyte system through a mediation by cytokines, potentially resulting in an elevated ferritin level and decreased serum iron (3). In areas with high malaria endemicity, availability of free serum iron may be picked up by *P. falciparum* with potential deleterious consequence of increased infection (38). We observed a significant reduction in the prevalence of underweight infants after adjusting for inflammation. The odds of infection obtained using reported symptoms differ widely from that of elevated acute phase proteins. Hence, adjusting for inflammation should also be considered when assessing the anthropometric measures in children from a rural population with a high prevalence of infection. There is potential suppression of food intake, impairment of nutrient absorption, and increased nutrient requirements during the acute phase response at the onset of infection (11). In fact, a decreased intake of both macro- and micronutrients have been associated with infection-related caloric suppression (11, 39, 40). Furthermore, during episodes of both enteric and non-enteric infections such as diarrheal diseases or helminthic attacks, absorption of nutrients can be greatly reduced (11) due to damage caused by the gut epithelial membrane cells (39–41). During febrile infection, there is direct loss of macronutrients such as proteins (proteinuria) and micronutrients such as retinol bound to RBP (41). This results in increased daily requirements of these nutrients by children (11). Also, infection by malaria and tissue damage from parasitic infection may culminate in blood loss and iron-deficient anemia through reduced iron absorption or iron loss in urine after hemolysis and sequestration of iron in macrophages of the mononuclear phagocyte system (18).

A study assessing the increased risk of respiratory disease and diarrhea among Indonesian pre-school-aged children with mild vitamin A deficiency found that children with stable vitamin A deficiency had increased risk of both respiratory disease and diarrhea. It also showed that the rates of diarrhea and respiratory disease among children with improving vitamin A status were the same as those of children who did not have vitamin A deficiency (42, 43). Another study in Thailand did also report greater risk of respiratory disease in mild vitamin A-deficient children (44). After adjusting for the intervention group and other reported illnesses, we found that the odds of having diarrhea among vitamin A-deficient infants was 19% higher than the odds of those with no diarrhea. However, the association was not significant. But among those with respiratory infections, the odds of being vitamin A deficient were significantly higher than the odds of those who without vitamin A deficiency (adjusted *OR* = 1.75; *P* = 0.015).

A limitation of the study was that we did not assess body fat, a potential confounder of inflammation. The CF estimated in this study may not be generalizable or applicable to other populations with different levels of severity and frequency of infections and malnutrition status (18).

From our findings, we conclude that acute phase proteins are more useful in defining infection in a population for research purposes than reported symptoms of illness. Not controlling for inflammation in a population while assessing the nutritional status will usually result in inaccurate estimate of true population prevalence, likely different for different micronutrient indicators, as we have shown for vitamin A vs. iron markers.

## Data availability statement

The original contributions presented in this study are included in the article/supplementary material, further inquiries can be directed to the corresponding author.

## Ethics statement

The studies involving human participants were reviewed and approved by the Institutional Review Boards at Emory University and the Kenya Medical Research Institute. Written informed consent to participate in this study was provided by the participants’ legal guardian/next of kin.

## Author contributions

FG, AG, RW, JL, and DC designed the research and analysis strategy. FG, RW, RA-A, AG, HO, and RW analyzed the data and interpreted the results. FG, AG, HO, and RW supported the data collection. FG wrote the first draft of the manuscript and led further manuscript revisions, and had primary responsibility for the final content. All authors provided inputs on drafts and read and approved the final manuscript.

## Abbreviations

AGP: α -1-acid glycoprotein
ANC: antenatal care
CHW: community health worker
CRP: C-reactive protein
IYCF: infant and young child feeding
Mama SASHA: Sweetpotato Action for Security and Health in Africa
OFSP: orange-fleshed sweet potato
PLW: pregnant and lactating women
PNC: postnatal care
RAE: retinol activity equivalent
RBP: retinol-binding protein
VA: vitamin A
WDDS: women’s dietary diversity score.
